# Home Health Registered Nurses’ Experiences With Financial Exploitation of Older Women With Cognitive Impairment

**DOI:** 10.1093/geroni/igaa057.2460

**Published:** 2020-12-16

**Authors:** Carol Rogers, Lisa DeSpain, Janet Wilson

**Affiliations:** 1 University of Oklahoma Health Sciences Center, Reynolds Center for Geriatric Nursing Excellence, Oklahoma City, Oklahoma, United States; 2 University of Oklahoma Health Sciences Center, Oklahoma City, Oklahoma, United States

## Abstract

Older adults diagnosed with cognitive impairment (CI) who live at home are at high risk for FE due to dependence on caregivers and diminishing cognitive and financial capacities. Health care providers are mandated reporters for elder abuse, that includes financial exploitation (FE), one of the seven types of older adult maltreatments. Twenty Home Health Care Nurses (HHRN) of older adults in Oklahoma were interviewed to discover their understanding and experiences with FE. Transcripts were analyzed by conventional content analysis. Line-by-line codes were generated inductively and codes were grouped into categories and themes until data saturation was reached. Five themes emerged: Red Flags, Familiar Offenders, Dire Consequences, Barriers/Facilitators, Doing Better. Conclusions: HHRNs are an untapped resource to provide suggestions for improvements of FE detection/reporting of older adults with CI and to help formulate policies, procedures, strategies to improve coordination and communication among healthcare, law enforcement, and social service systems.

